# The Use of Some Clay Minerals as Natural Resources for Drug Carrier Applications †

**DOI:** 10.3390/jfb9040058

**Published:** 2018-10-19

**Authors:** Marina Massaro, Carmelo Giuseppe Colletti, Giuseppe Lazzara, Serena Riela

**Affiliations:** 1 Dipartimento di Scienze e Tecnologie Biologiche, Chimiche e Farmaceutiche (STEBICEF), University of Palermo, Viale delle Scienze, Ed. 17, 90128 Palermo, Italy; carmelogiuseppe.colletti@unipa.it; 2 Dipartimento di Fisica e Chimica (DiFC), University of Palermo, Viale delle Scienze, Ed. 17, 90128 Palermo, Italy; giuseppe.lazzara@unipa.it

**Keywords:** clay minerals, drug carrier, halloysite

## Abstract

The goal of modern research is to use environmentally preferable materials. In this context, clay minerals are emerging candidates for their bio- and ecocompatibility, low cost and natural availability. Clay minerals present different morphologies according to their layer arrangements. The use of clay minerals, especially in biomedical applications is known from ancient times and they are regaining attention in recent years. The most representative clay minerals are kaolinit, montmorillonite, sepiolites and halloysite. This review summarizes some clay minerals and their derivatives for application as nanocontainer for biologically active species.

## 1. Introduction

Clay minerals are a well-known class of compounds which have been used for pharmacological applications since ancient times. In prehistory, clay minerals were used for the treatment of minor ailments including infections, pains, aches, and food poisoning. In this context, several documents [1,2] indicate that *Homo erectus* and *Homo Neanderthalensis* usually used mixtures of clay minerals to treat wounds, while, in ancient Greek, they were used as antiseptic materials for skin affliction treatments, cicatrizes or to cure snake bites.

The medicinal use of clay minerals became more and more prominent when, during the Renaissance, the Pharmacopoeia classified clay minerals as drug. Up to now, clay minerals are widely employed in the pharmaceutical industry as common additives. They are for example used as oral treatment of diarrhea or as gastrointestinal protector; or for topical dermatological applications [3,4,5]. Besides, they have found application as diluents, lubricants, flavor correctors, carriers of active ingredients in pharmaceutical products and so on [1,4,6].

The formation of clay occurs mainly in geological environment following the action of atmospheric agents on other silicate minerals giving rise to layer-type aluminosilicate [7].

The arrangement of atoms in the structure gives these minerals a platy morphology; the further arrangement of the tetrahedral and octahedral sheets allows the classification of clay minerals in three categories: 1:1, 2:1 and 2:1:1. The phyllosilicates such as kaolinite and halloysite 1:1 have per each clay layer one tetrahedral and one octahedral sheet. The 2:1 clay minerals are made up of one octahedral sheet between two tetrahedral ones. In this class of clay minerals belong montmorillonite, laponite, and illite.

Finally, phyllosilicates such as cloisite, composed of 2:1:1 succession of the sheets, consist of a succession of the octahedral sheet adjacent to a 2:1 layer. The different chemical composition and structure usually cause clay minerals to possess structural or surface charge which allow properties such as swelling or cations exchange capacity. The first one is permanent and exists due to ion substitutions, whereas the latter usually depends on the pH value [8].

**Figure 1:**
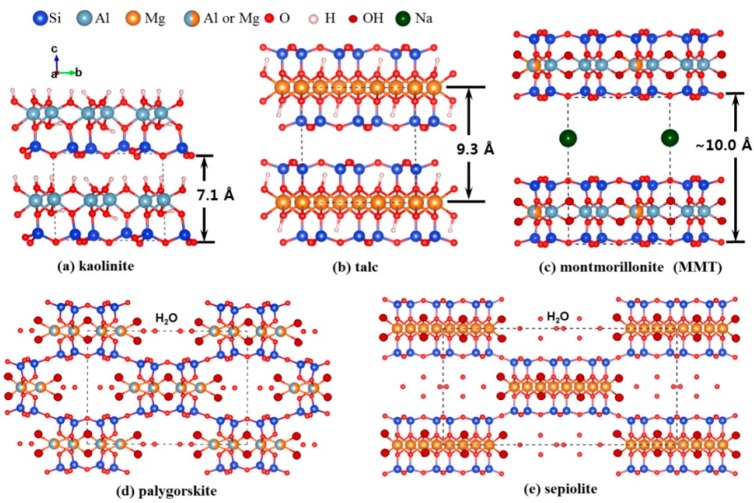
Crystal structure of the pharmaceutical used clay minerals: (**a**) kaolinite; (**b**) talc; (**c**) montmorillonite; (**d**) palygorskite; and (**e**) sepiolite. Dashed lines represent the unit cell. Reproduced with permission from [12].

Currently, kaolin (Si/Al oxide, 1:1), montmorillonite and sepiolite (Si/Al oxide, 2:1), where the 1 nm-thick clay aluminosilicate sheets are stacked in bulky platelets or processed with exfoliation to bilayer sheets (Figure 1), are the most used clay minerals for the research and applications for medical purpose. More recently, another clay mineral has gained attention, the allophane one (Si/Al molar ratio of 0.5 and 1, corresponding to Al-rich and Si-rich allophane, respectively) [9]. Commonly, allophane appears as nano- or micro-aggregates (clusters) differing in size and shape [9]. These characteristics endow allophane with abundant porosity and high specific surface area. In addition, allophane clay does not show any cytotoxicity effects [10] and can be used as carrier system for biological applications. Indeed, allophane has been used as carrier for cisplatin to minimize the drug side effects for application in anticancer therapy [11].

**Figure 2:**
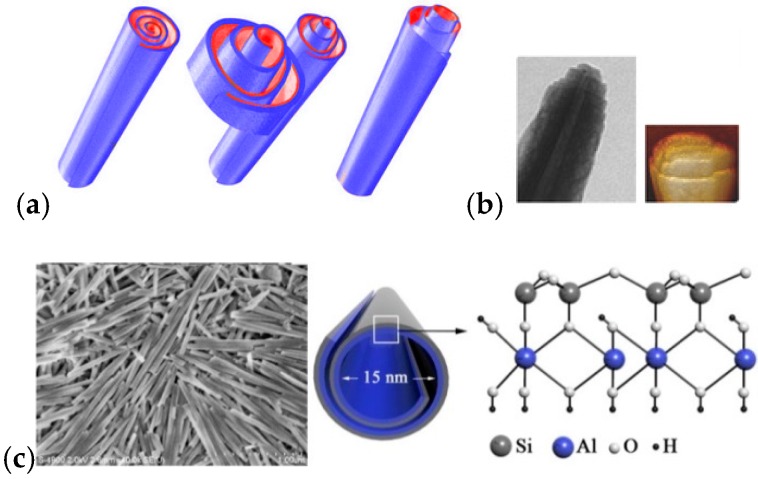
(**a**) Schematic representation of the rolled structure of halloysite. (**b**) Transmission electron microscopy (TEM) and atomic force microscopy (AFM) images of HNT edges. Reproduced with permission from [13]. (**c**) FE-SEM image of halloysite on Si-wafer (Left) and schematic illustration of crystalline structure of halloysite (Right). Reproduced with permission from [14].

Halloysite (Si/Al oxide, 1:1) is a clay mineral of the kaolin group with particular properties due to their tubular morphology (HNT) that is caused by the intercalation in interlayer region of a layer of water molecules (Figure 2).

**Figure 3:**
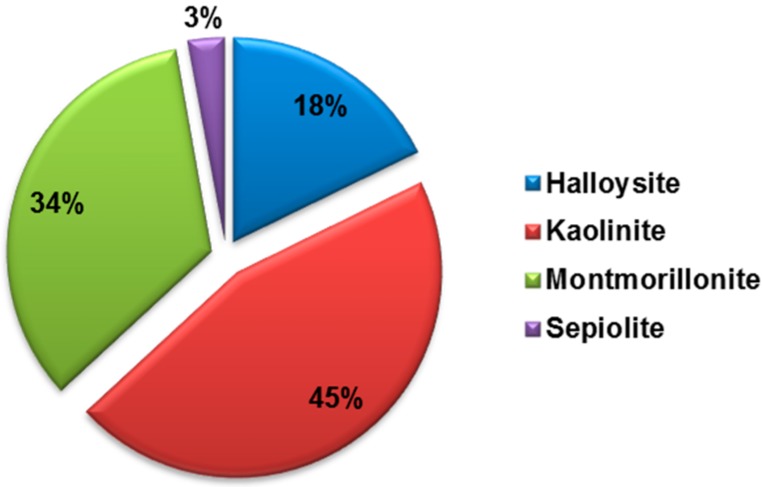
Comparison of the number of scientific publications on “Halloysite”, “Kaolinite”, “Montmorillonite” and “Sepiolite” refined each with the term “drug”. Data analysis of publications, as of June 2018, was done using the SciFinder Scholar search system.

The wide use of clay minerals is mainly due to their versatile properties such as high adsorption capacity and specific surface area, ion exchange capacity, colloid and thixotropy, chemical inertness swelling property, and, above all, low toxicity for oral administration. Among the plethora of clay minerals, the most used for pharmacological applications are kaolinite, talc, smectites (montmorillonite, saponite, and hectorite), palygorskite, and sepiolite [5,15,16] (Figure 3). Palygorskite, sepiolite, and smectites possess the peculiarity to adsorb protons in gastric acid and release non-toxic ions such as Mg^2+^ and Al^3+^ and therefore they might act as antacids by reducing the gastric acidity. Their physicochemical features have been also exploited for application as gastrointestinal protectors since they can efficiently increase the barrier thickness and, due to adhesion on to intestinal and gastric membrane, clay minerals decrease the gastric secretion and irritation.

Other uses are related to the application as antidiarrheals in which they can absorb the enteropathogens and reduce the liquid in the colon. Although their use in pharmaceutical formulation is advantageous, there are some risks associated to a prolonged administration of clay minerals. The long-term oral administration caused kidney stone formation [17,18,19] and the adsorption of some nutritive elements and enzyme, leading to their elimination from the body [5,20].

Several formulations based on clay minerals for dermatological applications have been developed, since clay minerals, such as kaolinite, talc, and smectite, show nonfibrous morphology and high adsorption capacity.

Besides the pharmaceutical application, clay minerals have been extensively used as excipients in some formulation; as lubricants in manufacturing pills; disintegrants; anticaking and thickening agents; binders and diluents; emulsifiers; and carriers of biologically active molecules for improving drugs bioavailability.

However, some limitations hinder the use of clay minerals as they are, because of the strong interactions which can exist between clay mineral and drugs that may influence the rate release of the drug, and therefore could limit the molecule bioavailability. This aspect represents a problem when drugs such as antihistamines are associated with clay minerals since they need an immediate therapeutic concentration in the blood. To overcome all these drawbacks, several attempts have been made over the years, and the most successful strategy is to functionalize the carrier. For example, one approach consists of coat the drug–clay hybrids with cationic polymers to slow down the drug release and ensure a prolonged administration of the drug over time.

This review summarizes the development in the use of clay minerals in the pharmaceutical field, focusing on kaolinite, montmorillonite, sepiolite, and halloysite. In particular, the clay mineral properties, their application as drug carriers and their functionalization are discussed.

## 2. Kaolinite

The kaolin group comprises predominantly kaolinite; thus, kaolin is usually wrongly used to replace the more accurate one of kaolinite, a 1:1 clay mineral, with an ideal formula of Al_2_Si_2_O_5_(OH)_4_, featured by a basic unit of a two-dimensional layer of silicate groups bonded to a layer of aluminate groups.

**Figure 4:**
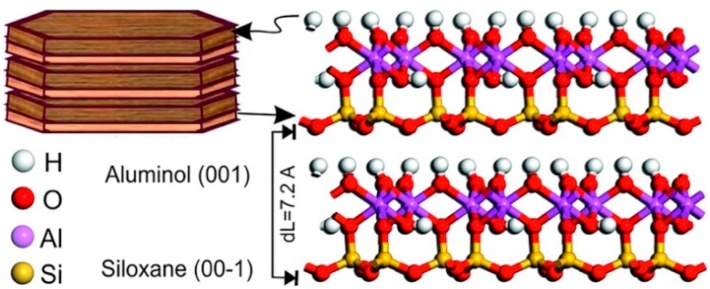
Molecular simulation model of kaolinite structure (1 × 2 × 2 unit cells) showing siloxane and aluminol surfaces. Reproduced with permission from [23].

The interlayer space of 1:1 clay mineral is about 7.2 Å, a value lower than the 2:1 clay mineral. It is little expandable due to the strong hydrogen bond in the interlayer space, allowing only few water molecules or cations to enter the structure (Figure 4). The more available surface is the external one, with an overall surface area limited to 10–30 m^2^ g^−1^ [21,22].

Furthermore, its cation exchange capacity is very low, which explain the low fertility of soils that are rich in kaolinite minerals.

In recent years, kaolinite is extensively used in several pharmacological preparations where it can be considered both an excipient which helps in the controlling the efficiency of the dosage form, and as an active component. In addition, in a kind of formulation, the presence of kaolinite can improve the drug bioavailability. If the kaolinite is used as excipient, it has to meet some important requisites: it must possess high chemical purity, keep the stability and consistency of its final dosage forms, and improve bioavailability and controlled release of active components in drug administration and delivery. In this context, it is used as diluent; binder; disintegrant; and pelletizing, granulating, amorphizing, particle film coating, emulsifying and suspending agents (Table 1).

Besides the commonly known properties, such as sorption capacity, high specific area, favorable rheological and swelling properties, inertness, etc., the kaolinite clay can induce and accelerate blood clotting. Thanks to this feature, it has been used as activating agent for clotting in physician’s routine practices and, from 2013, it was recognized by FDA as additive in ordinary gauze due to its efficiency in hemorrhage control without risk of thermal injury (it is commercialized as QuikClot Combat Gauze).

**Figure 5:**
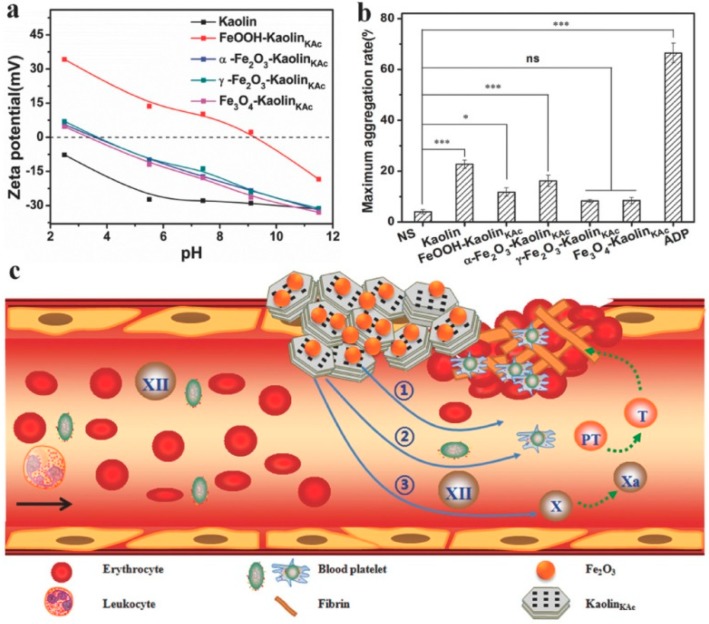
(**a**) ζ–potential curves of kaolin, FeOOH–kaolin_KAc_, α–Fe_2_O_3_–kaolin_KAc_, γ–Fe_2_O_3_–kaolin_KAc_, and Fe_3_O_4_–kaolin_KAc_. (**b**) Platelet aggregation induced by the samples, normal saline, and ADP represented as the maximal percentage aggregation of sodium citrated treated whole blood. (**c**) Illustration of the presumed synergism of α–Fe_2_O_3_–kaolin_KAc_ composite in hemostasis. Reproduced with permission from [49].

Recently, Yang et al. [49] developed an iron oxide–kaolinite nanocomposite (α–Fe_2_O_3_–kaolin_KAc_) for hemorrhage control. The authors used a combined approach where they exploited the properties of α–Fe_2_O_3_ and kaolinite to assemble a composite with excellent wound healing performances (Figure 5). In vivo tests show there exists a synergism between kaolinite and α–Fe_2_O_3_: the former absorbed fluid to concentrate blood platelets, RBCs, and clotting factors and activated the intrinsic coagulation cascade, whereas the latter facilitated RBC aggregation and clotting.

Similarly, Wang et al. [50] synthesized a new hemostatic sponge with kaolin powders embedded into graphene sheet. The authors found that the obtained sponge can stop bleeding in approximately 73 s in rabbit artery injury model. This outstanding result is also due to the kaolinite embedded in the graphene sheets. The clay indeed will activate hemostatic factors, such as Factor XXII, Factor X, Factor V, and platelets, favoring the hemostasis process.

The modification of kaolinite clay opens up several strategies to load and release in an efficient way different drugs. For example, by functionalization of kaolinite with a cationic surfactant, Li et al. [51] observed an increase in the loading of diclofenac. The introduction of the organic molecule, indeed, increases the clay mineral surface hydrophobicity and confers, to the clay, a positive surface which better interaction with the drug.

## 3. Montmorillonite

**Figure 6:**
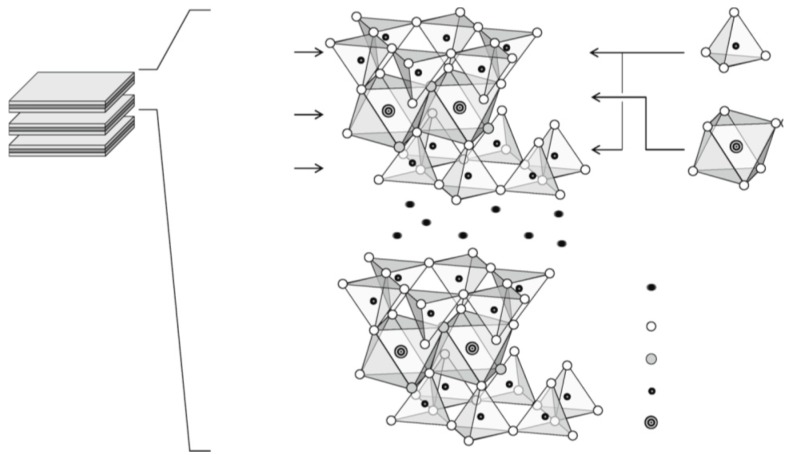
Molecular structure of montmorillonite. Reproduced with permission from [53].

Montmorillonite (MMT) is a dioctahedral 2:1 phyllosilicate constituted by two tetrahedral sheets (T:O:T) and one octahedral sheet. The main characteristic of montmorillonite is the interlayer space which is present between each triple-sheet-layer (Figure 6). Furthermore, the triple sheet layers consist of isomorphic substitutions in the tetrahedral sheet of Si^4+^ by Al^3+^ and Al^3+^ by Mg^2+^ in the octahedral ones. This arrangement confers to montmorillonite a negative residual charge compensated by cations in the interlayer space. The interlayer space constitutes about the 90% of the total clay surface and, thus, it confers to the mineral the possibility to absorb water molecule or other compounds, which increase the adsorption capacity of the clay for polar molecules [2,52].

Several studies have shown that MMT possesses low cytotoxicity both in vitro and in vivo. It has been demonstrated that this clay mineral does not possess any relevant cytotoxic effects in mice, even at the high dosage of 1000 mg kg^−1^. On the ground of these findings, MMT can be efficiently used as carrier system for several drug molecules.

According to the British, European and United States Pharmacopeias, the use of montmorillonite is mainly as auxiliary material in the pharmaceutical industry for topical or oral dosage forms. MMT indeed, thanks to its physicochemical properties (such as high swelling ratio), can intercalate active compounds between the layers generating a host for oral or topical drug delivery [54,55,56,57,58,59,60,61,62,63,64,65,66]. For montmorillonite topical use, it possesses beneficial effects in cosmetic and dermatological applications (paleotherapy and geotherapy) [1,67].

In this context, Iannuccelli et al. [68] developed a novel system based on organo-modified bentonite, for the topical delivery of gentamicin, which may represent a safer alternative and more effective way of gentamicin utilization. This drug possesses a low systemic absorption, probably due to the difficult penetration through the deep layers of the skin, and therefore its use is restricted to the local effect that involves mainly the most superficial skin layers. The authors highlighted that the drug was intercalated in montmorillonite clay, which favors its skin permeation, as shown by means of tape stripping test.

Turco Liveri et al. [69] reported the intercalation of metronidazole (MNE), an antibiotic commonly used for amoebiasis treatment, on MMT-K10 clay (a montmorillonite clay with a specific surface area of 250 m^2^ g^−1^). The authors found that the drug adsorption is strictly related to pH, and this process involves a multistep mechanism, where the drug can interact with the clay by protonation of the clay active sites, ionic exchange, and electrostatic interactions. Furthermore, the organic–inorganic hybrid composed by MNE/MMT-K10 allows for a sustained release of the drug in the gastrointestinal tract, protecting it by degradation, as highlighted by the in vitro kinetic study.

Praziquantel/montmorillonite complexes, with the drug intercalated in the montmorillonite interlayer space, were successfully developed by Viseras et al. [70]. To improve the bioavailability and the percent loading of the drugs into the clay, the supramolecular complex was obtained in a non-aqueous polar medium (ethanol). This strategy is promising alternative to improve bioavailability of such drugs that show low solubility in aqueous medium, such as praziquantel, since owing to the presence of the clay the dissolution rate of the drug has been increased. Montmorillonite was also used to improve the organoleptic properties of some drugs (see, for example, Sildenafil [18] and Aripiprazole [19]). Furthermore, the clay is useful to stabilize piroxicam, a common photolabile compound [20].

MMT has also been used to overcome the extrapyramidal side effects of chlorpromazine (CPZ) [71]. The intercalation of CPZ into the clay, indeed, is advantageous since the drug is released in a sustained manner for a longer period and, therefore, the side effects could potentially be reduced.

The effects of the functionalization of MMT with Tween-20 surfactant on the loading of cinnamic acid were evaluated by Sciascia et al. [72]. Adsorption isotherms of the drug on both MMT and MMT-Tween 20 hybrid confirm that the introduction of the non-ionic surfactant increases the adsorption rate of the cinnamic acid onto the clay.

**Figure 7:**
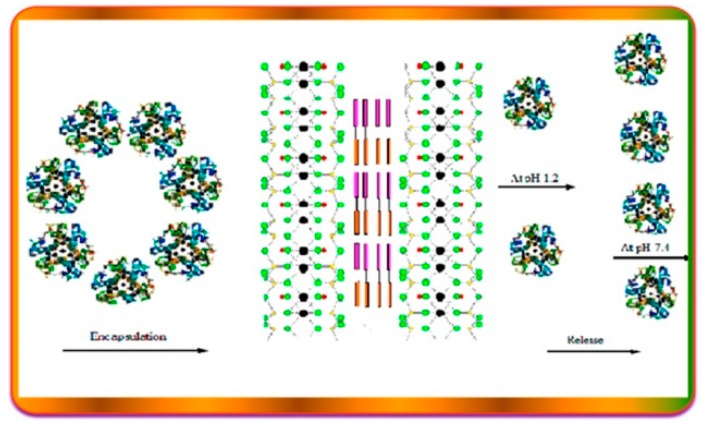
Schematic illustration for the release of Insulin from MMT clay functionalized with a thiolated chitosan–polyethylene glycol blend. Reproduced with permission from [73].

In another work [73], the MMT clay was functionalized with a thiolated chitosan–polyethylene glycol blend, obtaining a biodegradable composite for the insulin oral delivery. The authors found that this novel system limits the release of insulin in acidic medium (Figure 7), thus avoiding the protein inactivation in the gastrointestinal tract. Furthermore, biological in vitro studies on the carrier vehicle after the complete release of the protein show that it possesses antimicrobial activity, thereby making the system potentially biocompatible.

In this context, Ruiz-Hitzky et al. [74] incorporated, by an ion-exchange mechanism, metformin (MF), a drug usually used for the treatment of type 2 diabetes into Na-montmorillonite.

The physicochemical characterization of the hybrid MMT/MF shows that the intercalated metformin is arranged in the form of monoprotonated molecules disposed as a mono-layer covering the interlayer surface of montmorillonite.

The use of montmorillonite as a platform for the controlled release of MF could be advantageous to reduce the doses currently administered to patients with type 2 diabetes, to minimize side effects. Pure metformin hydrochloride, indeed, is commonly administered at high doses and produces collateral unsuitable gastrointestinal effects, which may be minimized with a better control of the drug administration.

In a composite system, constituted by MMT and polymers, drug molecules can interact both with the clay and the polymer matrix; therefore, it is interesting to figure out whether drug–polymer matrix or drug–clay interactions are more favorable. To answer to this, computational tools such as molecular dynamics (MD) and quantum chemistry are quite helpful. Tekin et al. [75] studied the interaction between curcumin, chosen as anticancer drug model, and a PLGA/MMT composite by means of different computational tools ranging from cluster, periodic DFT and MD simulations. All methods showed that curcumin strongly interacts with both PLGA and MMT and the presence of MMT is useful since it makes easier the release of curcumin due to the increased diffusion rate.

Finally, MMT was also used to develop an ecologic and economic fertilizer system, for the slow release of urea. To do this, several approaches are currently present in the scientific literature [76].

Pereira et al. [77] reported the exfoliation of MMT clay into urea matrix, by extruded methods. Conversely, Wanika [78] could efficiently intercalate urea in the MMT by a simple immersion of the clay in urea solution.

## 4. Sepiolite

The most used clay minerals for pharmacological applications are the ones derived from montmorillonite, even if, in the last years, sepiolites have attracted increasing attention since they lead to an improvement of the properties of biodegradable polymers [79].

**Figure 8:**
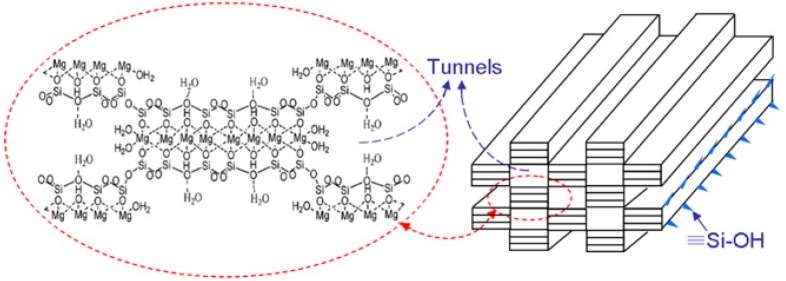
Crystalline structure and needle structure of sepiolite. Reproduced with permission from [82].

Sepiolite (Si_12_O_30_Mg_8_(OH)_4_(H_2_O)_4_ × 8H_2_O) is a naturally occurring fibrous clay mineral constituted by fine microporous channels, of 3.5 × 10.6 Å² in cross section [80], running parallel to the length of the fibers. The structure is in some aspects similar the common 2:1 trioctahedral silicates, but it is different from, for example, montmorillonite since it possesses channels due to inversion and discontinuities of the silica sheets (Figure 8). Some of the Si atoms at the corners of the outer blocks are bound to hydroxyls (Si–OH) [81].

Turco Liveri et al. [83] loaded Vitamin A (VitA) in Sepiolite (SPT) and MTM by impregnation. The studies of in vitro release kinetics showed that the release of VitA is related to the type of support as well as on the pH. Besides the controlled release, it was demonstrated that the SPT is able to prevent the oxidative degradation of the VitA [83].

Sepiolite clay was efficiently dispersed in gelatin-egg white biodegradable films for active food packaging applications by Guimerez et al. [84]. The introduction of the clay mineral showed a remarkable enhancement in the films mechanical properties and, after incorporation of clove essential oil, an improvement of their antimicrobial and antioxidant activities was observed. The authors explained these results by a clay effect on the controlled release of eugenol from the film, by hindering the interactions between the protein polymeric matrix and the essential oil.

Chitosan/Poly vinyl alcohol (PVA)/sepiolite hybrid hydrogel films were prepared for the loading of cefazolin [85]. The hydrogels were made by crosslinking chitosan with PVA by means of freezing–thawing cycles. Afterward, to this, sepiolite was added as reinforcing agent, and it was used to load and release the drug. The antibacterial property of the novel films against gram-negative and gram-positive bacteria was finally studied. The cefazolin-loaded films showed wider area of inhibition against *Bacillus cereus* bacterium.

Another work reports the use of sepiolite for the adsorption and release of nitric oxide (NO), an endogenous molecule with an important biological action [86]. The toxicity of the sepiolite/NO hybrid was investigated towards HeLa cells and the obtained results showed a high survival rate, even for material concentrations higher than 450 mg mL^−1^ mainly due to the use of materials that are prepared from a naturally occurring clay. These findings assert the sepiolite can be used, similar to other universally recognized clay, for biological applications since it ensures low toxicity at higher doses.

Sepiolite toxicity was investigated by several research group. Sepiolite, with its microfibrous structure, has raised safety concerns, since the SPT fibers can stick in the human body after their adsorption, causing the same damages of asbestos. The SPT safety was thoroughly investigated by Ruiz-Hitzky et al., who showed that some deposits of sepiolite reported previously [87] possess the optimal dimensions to be excluded by cells without toxicity. Therefore it is possible that sepiolite may pose a low health risk, especially when it possesses fiber with a <5 mm length [88]. Based on the aforementioned features, the International Agency of Research on Cancer (IARC) has classified the sepiolites as non-hazardous and non-carcinogenic material [88].

## 5. Halloysite Nanotubes

Halloysite nanotubes (HNTs), with chemical formula Al_2_Si_2_O_5_(OH)_4_·nH_2_O, are 1D natural inorganic clay nanotubes belonging to kaolin group. It is a dioctahedral 1:1 clay mineral present in soils especially in wet tropical and subtropical regions and weathered igneous and non-igneous rocks.

Each deposit is characterized by different purity grade, characteristic sizes, and hydration state.

The name of “halloysite” is due to a mineralogist, M. Berthier, who first used it in 1826 in honor of Omalius d’Halloy, who found the mineral in Angleur, Liége, Belgium.

**Figure 9:**
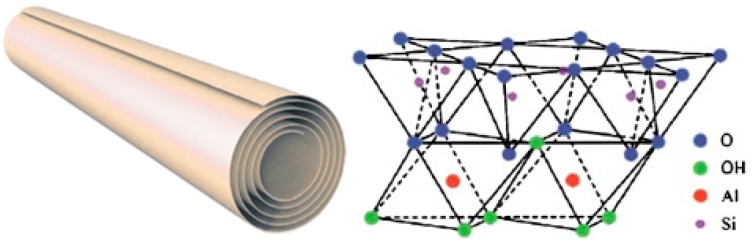
Schematic representation of the rolled structure of halloysite (**left**) and schematic illustration of the crystalline structure of halloysite (**right**). Reproduced with permission from [89].

Typically, the halloysite clay possesses a hollow tubular structure in the sub-nanometer range with an aspect ratio of ca. 20; the wall is constituted of 10–15 bilayers, with a spacing of approximately 0.72 nm and has a density of 2.53 g/cm^3^. The external surface is composed of siloxane (Si–O–Si) groups, while the inner one consists of a gibbsite-like array of aluminol (Al–OH) groups, and Al–OH and Si–OH groups at the edges of the tube. The sequence of the layers gives to the tubes Si–O groups at the outer surface and Al–OH groups at the inner surface (Figure 9) [89,90,91]. At pH 3–8 [92], the HNT surfaces are oppositely charged according to the different chemical composition of their groups which undergo ionization [93].

The halloysite length is generally in the range of 0.2–1.5 µm, while the inner and outer diameters of tubes are in the ranges of 10–30 nm and 40–70 nm, respectively [94,95,96], depending on the extraction site and purification processes. In some deposits, halloysite tubes with length up to 3–5 μm were found but in the size distribution curve they have a minor fraction. However, these shorter tubes are the most attractive from a biological point of view since they are more suitable for composites with sustained delivery of chemicals and drugs in comparison to the longer ones. Besides, clay tubes 1 μm in length may have an additional advantage because it is a safe dimension for macrophage removal of the nanoparticles from living organisms [96].

**Figure 10:**
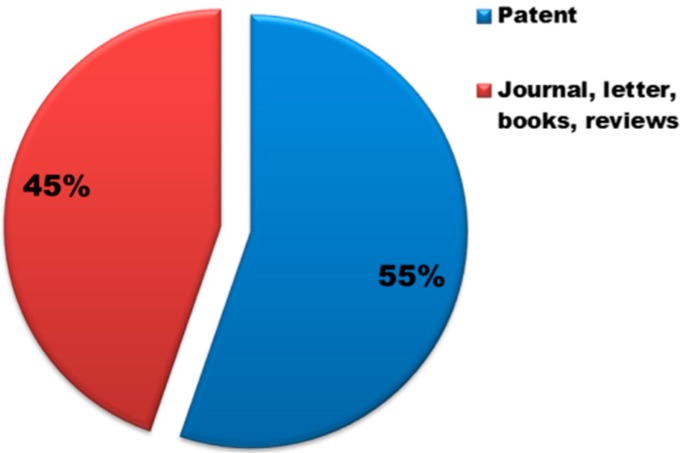
Distribution (%) of scientific publications in “patent” and “journal” for halloysite. Data analysis of publications, as of June 2018, was performed using the SciFinder Scholar search system using as “Document type” the “Journal, letter, book, reviews” and “Patent”, respectively.

In the past, the main use of this clay mineral had been only in ceramics, as alternative to kaolinite. Since 2005, however, with the growing interest in biomedical clay materials, several uses of the clay have been taken into account. The increasing interest in the halloysite is testified by the number of patents which has virtually equaled the number of publications on the subject (Figure 10). Based on these findings, the interest in the industry seems evident which can be translated into important new technologies that could in the future become more prominent with respect to other clays.

The most interesting aspect of the halloysite clay is its different surface chemical composition which allows the selective modification leading to the synthesis of different appealing nanomaterials.

The modification of the halloysite lumen is mainly based on the electrostatic interactions between negatively charged molecules and the positive aluminum surface. Pioneering work by Lvov, Price, and Veerabradan highlighted the possibility to include in the lumen drug molecules such as tetracyclines [97]. Since then, many different molecules have been successfully loaded on halloysite lumen, which benefits of a sustained release over the time and improved biological activity [97,98,99,100,101,102,103,104].

**Figure 11:**
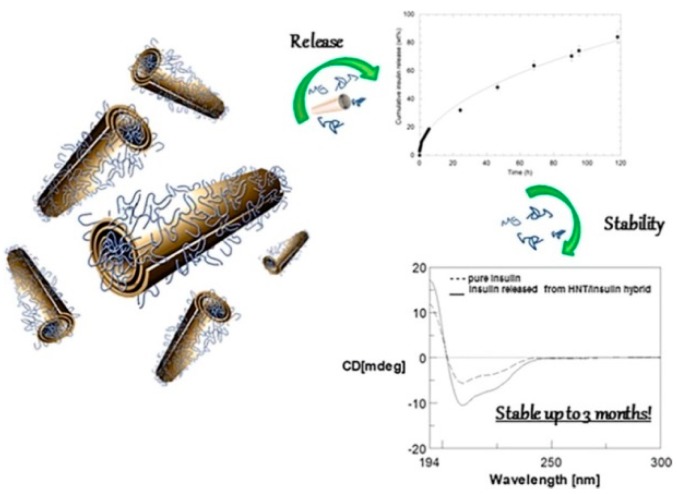
Schematic representation of the HNT/insulin hybrid, release, and stability. Reproduced with permission from [106].

Insulin, a protein which shows several drawbacks for its oral administration, such as rapid degradation, digestion, and inactivation by proteolytic enzymes [105], was efficiently loaded on HNT nanomaterial by exploiting the different surfaces charge of the clay [106]. It was found that the maximum loading is achieved in acidic medium, where the protein can interact mostly with the HNT lumen and also with the external surface as verified by ζ–potential measurements. The HNT/insulin hybrid presents a more negative ζ–potential (−45.9 mV) compared to that of pristine HNTs (−34.4 mV), in agreement with the selective electrostatic interactions between the positive halloysite inner surface and insulin. The loading of insulin into HNT led to the synthesis of an efficient nanocarrier which allows a prolonged and sustained release of the protein over seven days. Noteworthy, dichroism circular experiments highlight that the protein is released from the carrier in its native form (Figure 11).

Similarly, Lvov et al. immobilized on halloysite surfaces some proteins with globule diameters of 3–8 nm by means of electrostatic interactions [102]. Therefore, proteins such as laccase, lipase, glucose oxidase, and pepsin can be efficiently immobilized both in the lumen and on the external surface exploiting the different surface charges.

The different charges are important for drug delivery since they allow closing the halloysite edges with suitable molecules. In this way, it is possible to slow down the release of a drug from the lumen. In this context, Fakhrullin et al. reported the immobilization of brilliant green into HNT followed by coating the hybrid complex with dextrin [107]. The choice of similar coating is due to the fact that it can be disrupted once the cell absorbs these nanocarriers where sugar can be cleavable by intracellular glycosyl hydrolases. Due to the presence of dextrin, the drug may be preferentially released in the rapidly proliferating cells, such as tumor cells, which are prone to internalizing the nanotubes. Duce et al. used HNT as carrier for salicylic acid (SA) for application in the field of active packaging for food industry [108]. The authors thoroughly investigated the hybrid HNT/SA from a physicochemical point of view demonstrating its ability to stabilize HNT suspensions. The biological properties of the hybrid were investigated by studying the antibacterial properties of the SA against Pseudomonas fluorescens IMA 19/5, highlighting an actual application as packaging for food protection.

However, the application of pristine halloysite as nanocontainer, despite the several examples reported, is limited. Halloysite clay can establish on weak interaction with guest molecules, based on hydrogen bonds or Van der Waals forces, resulting in low capability for tunable drug release [52,109].

The covalent modification of the tubes with suitable moieties is a good strategy adopted to solve these drawbacks.

For example, the external halloysite surface is efficiently covalently modified by grafting organosilane molecules via condensation with the hydroxyl groups of the edges or the defects of the surface. Owing to this, different materials of large interest were obtained with consequent increase of the HNT application fields [106,110].

Among the different organosilane molecules which can be used for the HNT modification, the most used one is for sure the 3-aminopropyltriethoxysilane (APTES). Due to the introduction of an amino group on the HNT external surface, the modification allows obtaining a material that can be further modified and therefore with interesting feature for application in the drug carrier and delivery field.

For example, Kumar-Krishnan et al. used the HNT–APTES compound for enzyme loading, finding that glucose oxidase, for example, is better loaded on halloysite than p-HNT without loss of its biocatalytic performances. Noteworthy, the functionalization avoids the enzyme partial deactivation [111,112].

A similar strategy was used to increase, with respect to pristine halloysite, the ibuprofen (IBU) loading. The electrostatic interaction between the amino groups on the surface of the tubes and the carboxyl groups of IBU allowed to obtain better loading efficiency and also slows down the kinetic release of the drug, improving the performance of the material [109,113].

Similar results were obtained by Yang et al. immobilizing aspirin molecules on HNT–APTES. The authors found an increase in the drug loading, from 3.84 to 11.98 wt% compared to pristine halloysite. Furthermore, once again, the kinetic release of the drug was slowed down compared to the similar system based on pristine halloysite [114].

Halloysite–cyclodextrin nanosponge hybrid was used for the loading of clotrimazole (CLT), an antifungal drug commonly used for the buccal or vaginal treatment of candidiasis [115]. The HNT carrier was developed ad hoc by the covalent grafting of β-cyclodextrin on the HNT external surface [116]. In this way, a multicavity system was obtained which has shown enhanced loading performances. Once synthesized, the hybrid was further functionalized by cysteamine hydrochloride grafting to confer mucoadhesive properties to the overall system. Release studies showed that the hybrid materials reported in this study avoid the deactivation of CLT by preserving it from the hydrolysis of the imidazole ring.

A similar approach was exploited to attach on the HNT external surface some cyclodextrin units (HNT–CDs) [117]. The obtained multifunctional carrier presents an enormous advantage with regard to the conventional carrier system; the two different cavities offer the remarkable possibility for a simultaneous loading of one or more drugs with different physicochemical properties, followed by different paths for drug release consistent with the different types of the cavities interacting with the molecules. To exploit this aspect, two different drugs were chosen as model: quercetin and silibinin, two flavonoids which possess different size and shape. The experimental findings show that the silibinin molecules interacts preferentially with the HNT lumen, while the quercetin with the cyclodextrin cavity. The different interaction site of the two drugs allows for a targeted release in different conditions. The in vitro release tests performed in physiological conditions by means of the dialysis membrane method showed that silibinin was better released in acidic solution, and therefore in a medium simulating the gastric environment, while quercetin was better released in a medium simulating the intestinal fluid (phosphate buffer pH 7.4). Cytotoxic studies performed on 8505C cell lines highlighted that the drugs delivered by halloysite hybrids showed improved antitumoral efficacy compared to the free drug. Finally, the interaction between cells and the carrier, analyzed by fluorescence microscopy, showed that the materials were taken up into cells surrounded the nuclei.

If the same HNT–CDs system was further functionalized with some sugar moieties (such as mannose units, specific for binding cellular lectin) [118,119], an enhanced cellular internalization was observed, as revealed by fluorescence microscopy, due to the carbohydrate-receptor-mediated endocytosis. In this case, the nanomaterial is mainly localized in the cell nucleus.

A thermo-responsive polymer, such as poly-(*N*-isopropylacrylamide) (PNIPAAM), was grafted on HNT external surface by amide condensation mediated by DCC [120]. This nanomaterial presents some interesting features: (i) an empty HNT lumen, which can encapsulate biologically active molecules; and (ii) the polymer brushes which can interact with a suitable drug. In addition, PNIPAAM shows a low critical solution temperature (LCST) around 32 °C, after which the polymer brushes collapse on the HNT surface and therefore they could allow a targeted release of a drug.

In this context, the interaction of HNT–PNIPAAM hybrid and curcumin was investigated. It was found that the curcumin molecules are released from the carrier only a temperature of 37 °C and mainly in a medium that mimics the intestinal fluid. Since the carrier retains the almost totality of the drug loaded in acidic medium, it represents a versatile drug carrier system for the targeted delivery of curcumin, preventing its degradation in the gastrointestinal tract.

An interesting alternative to obtain hybrid HNT–Curcumin hybrid systems is the direct covalent grafting of the bioactive molecule on the surface of the tubes developing a pro-drug system [121].

The prodrug so-obtained has shown specific properties; the covalently linked curcumin, indeed, can be released by a specific external stimulus, in particular, the kinetic release is influenced by both glutathione concentration or pH conditions. Therefore, in this work, a novel system with a dual stimuli-responsive nature is obtained. The potential application of the system can be also increased by further modification of HNT. The presence on an empty cavity, in the clay, allows to load other molecules in the lumen obtaining a prodrug system with could possess synergistic effects.

The synergistic effect between a drug loaded in the inner lumen and another covalently grafted on the external surface was also exploited by us [118]. In this context, we have synthesized, characterized and studied the antioxidant properties of HNT with complementary antioxidant functionalities based on Trolox and quercetin. Experimental findings showed that the Trolox molecules, covalently grafted on HNT external surface, were available to trap free radicals, whereas the quercetin, second co-antioxidant, supramolecularly linked on the lumen, were slowly released to generate prolonged synergistic protection.

Similar to the HNT–PNIPAAM thermo-responsive carrier, a synergistic effect was also observed in a pH-sensitive HNT–triazolium salt carrier [122,123]. As reported for the HNT–Trolox/Que system, in this case, the presence of the triazole moiety on the external surface could exert some synergistic effects with a drug loaded onto the halloysite surfaces, due to the biological properties of triazolium. Based on this evidence, the halloysite based triazolium salts were used as carriers for curcumin and cardanol. The physicochemical characterization showed that the electron rich molecules can interact with the positively charged halloysite lumen and besides with the external surface by π–π interactions between the aromatic rings of cardanol and curcumin and that of triazolium salts. The coexistence of these two species enhances the loading efficiency of the clay compared to that with pristine halloysite and an increased cytotoxicity towards different tumoral cell lines.

**Figure 12:**
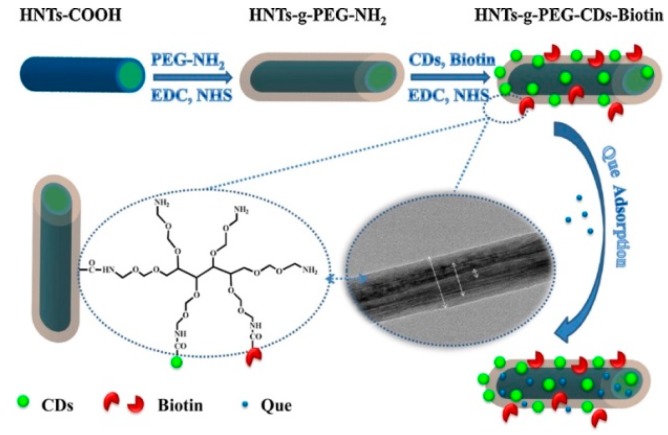
Schematic illustration of the chemical structure and preparation procedure of. HNTs-g-PEG-CDs-Biotin, followed by Que adsorption. Reproduced with permission from [124].

To enhance the loading of quercetin (Que) and obtain a slow release over the time of the drug, He at al. grafted on modified HNT, a six-arm (Poly Ethylene-Glycol) amine (HNTs-g-PEG) [124]. This system was further modified by biotin conjugation, to achieve a selective delivery into carrier cells. Finally, to image the Que delivery through the body, on the HNTs-g-PEG-Biotin were loaded fluorescent carbon dots (Figure 12).

MTT test showed that the obtained hybrid possesses a low cytotoxicity and excellent biocompatibility and, above all enhanced anti-tumor effects, when loaded with Que, against HeLa cells. HNTs grafted with CDs and Biotin nanoparticles on PEG have shown potential applicability as targeting carriers for delivery of drugs and in vitro and in vivo ability of could be tracked by the imaging.

By means of similar strategy, Liu et al. covalently conjugated the PEG polymer to prolong halloysite circulation time and controlling its dosing interval [125]. Conversely to the above example, in this case, to obtain tumor targeting systems, the authors covalently grafted folate units to the PEG functionalized halloysite. The HNT–PEG–FA hybrid was used as carrier of doxorubicin (DOX) for the treatment of breast cancer. The in vitro release test highlighted that DOX is released from the carrier up to 35 h in an acidic environment (pH = 5.3), while it is relatively stable in neutral conditions. Cytotoxicity assays showed that the system can restrain proliferation and induce death of FR+ MCF-7 cells, while it shows relative low cytotoxicity towards the FR^-^L02 cells. Furthermore, the DOX complex can produce more ROS in MCF-7 cells which leads to apoptosis.

The same authors used a similar approach to link on carboxylic acid functionalized halloysite, another polymer, specifically the chitosan one. This new system was efficiently used for the loading and release of doxorubicin and curcumin [126,127]. Since chitosan itself possesses interesting biological properties, the system developed in this work, showed good hemocompatibility, stability in body fluids and enhanced cytocompatibility. Furthermore, the modified halloysite allows in a targeted release; both drugs were released in their active form and in a sustained manner only in the “tumoral environment” instead of in the normal physiological conditions, avoiding in this way, possible side effects of the antitumoral therapy.

Chitosan is widely used in the biological field since it possesses anti-infection and hemostatic activities; due to these properties, a chitosan/HNT nanocomposite could be utilized for wound healing since it was found that it helps the re-epithelialization and collagen deposition.

More recently, several polymers were used for the HNT functionalization, obtaining different nanocomposite which have showed to be efficient in the delivery of drugs.

In this context, Fan et al. prepared halloysite–sodium alginate/hydroxyapatite nanocomposite beads by generation of hydroxyapatite (HA) in a nanosized regime [128]. On these beads was efficiently loaded the diclofenac sodium drug. Similarly, ofloxacin (OFL) was loaded on magnetic microspheres, 2-hydroxypropyltrimethyl ammonium chloride chitosan/Fe_3_O_4_/halloysite. Halloysite once again, is helpful in improving the OFL bioavailability in the gastrointestinal tract [129].

Interesting nanocomposites are those obtaining by the introduction of HNT filler in a hydrogel matrix. In this context, to increase the filler dispersion in a peptide hydrogel, we have modified the external HNT surface with amino acid derivatives. To reach this goal, starting from a HNT–NH_2_ nanomaterial, we covalently grafted the Fmoc–phenylalanine molecule (f–HNT) [130].

The presence of the amino acid functionality on the halloysite surface allows specific π–π interactions between modified halloysite and Fmoc-phenylalanine (Fmoc-Phe) generating a hybrid hydrogel with affected physicochemical properties which was efficiently used for the loading and release of camptothecin molecule (CPT).

CPT was also loaded on folic acid conjugated chitosan oligosaccharides assembled magnetic halloysite nanotubes (FA-COS/MHNTs) [131]. This hybrid showed excellent receptor-specific targeting effects for Caco-2 cells, high superparamagnetic properties, an outstanding usefulness in killing the cancer cells. Moreover, under acidic conditions, the FA-COS/MHNTs showed a sustained drug release up to 60 h.

All example reported showed that HNT is a promising material for biological applications since it can deliver a given species in a sustained and controlled manner, avoiding, in same case, its degradation and deactivation.

Unfortunately, the HNT application is limited to the oral administration since the human body does not have the biological mechanisms to efficiently dispose the aluminosilicate degradation. However, halloysite is a biocompatible material as testified by several studies.

It was found that the clay is not toxic towards several cell lines such as mammalian cells and some living organisms. In this context, the interaction of HNT with microscopic algae *Chlorella pyrenoidosa*, fresh water ciliate protist *Paramecium caudatum* [132] and *Escherichia coli* bacteria [128] as well as in yeast cells [94], was studied by Lvov and Fakhrullin et al. The obtained results highlight that no penetration of the nanomaterial into cell interior occurs [133] and that it is also not toxic in a wide range of concentration.

In vivo studies on living nematodes (worms), *Caenorhabditis elegance* [134], chickens and piglets have shown that, by feeding them with halloysite, there is no toxicity in the organism and in the case of superior organisms the clay can remove some mycotoxins potentially present in grain feed [135].

As far as halloysite accumulation after its oral administration is concerned, a recent study has shown by several investigations of target organs that HNTs are safe materials [136].

In this context, Hu et al. [137] demonstrated that the prolonged administration of the clay by an oral route up to 30 days can lead to an Al accumulation in the liver with consequent hepatic damage. Thanks to this study, it is possible to determine the maximum dose that can be safely used for HNT oral administration (ca. 20 mg kg^−1^ BW).

Finally, in a recent study, we also investigated the effect of halloysite on *Raphanus Sativus* L. to evaluate any potential phytotoxic effects. Our study highlights that it not only possesses no negative effects, but it seems that HNT can help the growth of the seeds [138].

## 6. Conclusions

Clay minerals have attracted great interest since they are biocompatible materials with appealing properties. The most representative examples of clay minerals are kaolinite, montmorillonite, sepiolites and halloysite. The present review reports the recent developments of the clay minerals research, focusing on application as carrier for the delivery and sustained release of biological active species. The functionalization of clays surfaces, by means of supramolecular interactions or covalent modifications, opens different ways to obtain interesting nanomaterials which show improved biological properties with respect to the unmodified ones.

## Figures and Tables

**Table 1:** Kaolinite functionalities as pharmaceutical excipient. Adapted with permission from [23].

Excipient Functionality	Kaolin Grade/Composites	Commercial/Excipient Dosage Form	References
Diluent	Light ^a^ and heavy ^b^ kaolin Heavy kaolin	Herbal slimming tablet: products (Slimwell^®^ and Quantrim^®^). Gastro-resistant tablets: Mecysteine Hydrochloride^®^ 100 mg. Granules for oral suspension: Riclasip^®^ and Co-amoxiclav DST Grünenthal^®^. Riboflavin (vitamin B2) hard gelatin capsules. Pyridoxine hydrochloride (vitamin B6) kaolin capsules. Thiamine (vitamin B1) and ascorbic acid (vitamin C) tablets and capsules.	[24,25,26,27,28]
Binder	Kaolin/Eudragit^®^ E30D mixture	Tablets and capsules	[29,30]
Disintegrant	Heavy kaolin	Tablets and pellets	[31,32,33]
Granulating agent	Heavy kaolin Light kaolin	Granules: (10% sodium chloride). Granules: (20% calcium chloride, with polyethylene glycol and polyvinyl alcohol).	[32,33,34,35]
Pelletizing agent	Chitosan/kaolin Heavy kaolin	Microcrystalline cellulose and hydrochlorothiazide (HCT) pellets. Pellets (5% sodium lauryl sulfate) of size range (850–1180 μm). Pellets (25% kaolin and 5% crospovidone). Pellets (kaolin with microcrystalline cellulose and lactose). Pellets (45% kaolin, 5% aerosil^®^ 200, 39.5% lactose, 2.5% liquid paraffin and 8% hydroxypropylmethylcellulose phthalate).	[36,37]
Amorphizing agent	Light kaolin	kaolin-ibuprofen solid dosage forms.	[38]
Film-coating additive	Koallicoat IR system and Kaolin/Eudragit^®^ E30D dispersion	- Hypericon^®^ and Metformin^®^ tablets. - Pseudoephedrine hydrochloride, theophylline, and diphenhydramine hydrochloride pellets. - Dyphylline^®^ coated tablets.	[39,40]
Wetting and emulsifying agent	Light kaolin	Sulfur ointment. Oil-in-water Pickering emulsions. Non-aqueous oil-in-oil emulsions.	[41,42]
Suspending and anticaking agent	Heavy kaolin	Toxiban^®^, Kaolin-Pectin^®^, Kapect^®^, Kaolin, and Morphine mixture BP^®^ suspensions.	
Drug carrier	Heavy kaolin and light kaolin Light kaolin Metakaolin Methoxy-modified kaolinite Kaolin and metakaolin	Kaolin powder (high and low kaolinite crystallinity) loaded by sodium amylobarbitone. Porous kaolin-based pellets loaded by Diltiazem HCl. Pellets loaded by highly potent opioids. The derivative powder loaded by anticancer 5-fluorouracil drug and herbicide amitrole. Powder loaded by α-lactalbumin, bovine serum albumin and β-lactoglobulin protein.	[43,44,45,46,47,48]

^a^ Light, white powder, free from gritty particles, odorless and unctuous to the touch. A native hydrated aluminum silicate, selectively sourced and dried and milled to a fine powder. It contains a suitable dispersing chemical and is tested for chemical and physical properties in the manner defined by the specification of the British Pharmacopoeia 2013. ^b^ Fine, white powder, odorless and smooth to the touch and practically insoluble in water and in organic solvents. A refined, natural hydrated aluminum silicate. It is tested for chemical and physical properties in the manner defined by the specification of British Pharmacopoeia 2013.
